# Immunotherapy and drug sensitivity predictive roles of a novel prognostic model in hepatocellular carcinoma

**DOI:** 10.1038/s41598-024-59877-9

**Published:** 2024-04-25

**Authors:** Xiaoge Gao, Xin Ren, Feitong Wang, Xinxin Ren, Mengchen liu, Guozhen Cui, Xiangye Liu

**Affiliations:** 1 Cancer Institute, Xuzhou Medical University, Xuzhou, 221002 Jiangsu Province People’s Republic of China; 2 Department of Oncology, Jiangyin Clinical College, Xuzhou Medical University, Jiangyin, 214400 Jiangsu Province People’s Republic of China; 3 Department of General Surgery, The Affiliated Hospital of Xuzhou Medical University, Xuzhou, 221002 Jiangsu Province People’s Republic of China; 4 School of Information and Artificial Intelligence, Anhui Agricultural University, Hefei, 230036 People’s Republic of China; 5 School of Bioengineering, Zhuhai Campus of Zunyi Medical University, Zhuhai, 519040 Guangdong Province People’s Republic of China; 6 Jiangsu Key Laboratory of Immunity and Metabolism, Department of Pathogenic Biology and Immunology, Xuzhou Medical University, Xuzhou, 221004 Jiangsu Province People’s Republic of China; 7 National Demonstration Center for Experimental Basic Medical Science Education (Xuzhou Medical University), Xuzhou, 221002 Jiangsu Province People’s Republic of China

**Keywords:** Hepatocellular carcinoma, Prognosis prediction, Immunotherapy response, Drug candidate, Hepatocellular carcinoma, Prognostic markers

## Abstract

Hepatocellular carcinoma (HCC) is one of the most significant causes of cancer-related deaths in the worldwide. Currently, predicting the survival of patients with HCC and developing treatment drugs still remain a significant challenge. In this study, we employed prognosis-related genes to develop and externally validate a predictive risk model. Furthermore, the correlation between signaling pathways, immune cell infiltration, immunotherapy response, drug sensitivity, and risk score was investigated using different algorithm platforms in HCC. Our results showed that 11 differentially expressed genes including UBE2C, PTTG1, TOP2A, SPP1, FCN3, SLC22A1, ADH4, CYP2C8, SLC10A1, F9, and FBP1 were identified as being related to prognosis, which were integrated to construct a prediction model. Our model could accurately predict patients’ overall survival using both internal and external datasets. Moreover, a strong correlation was revealed between the signaling pathway, immune cell infiltration, immunotherapy response, and risk score. Importantly, a novel potential drug candidate for HCC treatment was discovered based on the risk score and also validated through ex vivo experiments. Our finds offer a novel perspective on prognosis prediction and drug exploration for cancer patients.

## Introduction

In 2020, primary liver cancer is the third lethal cause of cancer-related deaths in the worldwide^1^. Most currently, a predictive study showed that 1.3 million people could die from liver cancer in 2040, which is a 56.4% increase compared to the number in 2020^2^. Therefore, liver cancer will be significant leading cause of deaths in the future. Prognosis prediction and drug treatment recommendation for liver cancer are mainly based on tumor burden, liver function, and physical status of cancer patients^3^. In recent years, the role of tumor biomarkers in diagnosis and prognosis for liver cancer has gradually been recognized. Satisfactorily, risk models established with tumor biomarkers have been used to predict survival, metastasis, or recurrence of cancer patients^4,5^. Therefore, it will be significant to develop a predictive risk model to predict survival of patients with hepatocellular carcinoma (HCC).

Compared to normal tissues, a series of genes exhibited differential expression in tumors, which act as oncogene or tumor-suppressor and play an essential role in the progression of cancer. Interestingly, a number of differentially expressed genes (DEGs) act as biomarkers to predict the survival of cancer patients. Currently, public datasets including the cancer genome atlas (TCGA), international cancer genome consortium (ICGC), gene expression omnibus (GEO), clinical proteomic tumor analysis consortium (CPTAC), human protein atlas (HPA), and genotype tissue expression (GTEx) have brought significant convenience to compare gene expression and achieve individual clinicopathological variables. Based on these datasets, several studies showed that DEGs could be used to develop prognosis risk model for predicting the survival of cancer patients^6–8^. Of note, these models could also be applied to evaluate the response of cancer patients to immune therapy and drug sensitivity^4,8,9^. Unfortunately, most studies mainly focused on oncogenes but ignored tumor-suppressors. For this reason, we integrated up- and down-regulated genes in HCC to develop a novel prognosis risk model predicting survival of cancer patients.

In this work, we primarily integrated four oncogenes and seven tumor-suppressors to develop a prognosis risk model based on TCGA dataset. Furthermore, the correlation between signaling pathways, immune cell infiltration, immune checkpoint inhibitors, and risk score were characterized in HCC individuals. Subsequently, the response of HCC patients to immunotherapy was also assessed based on the risk score. Moreover, a cluster of six drug candidates were screened for HCC, their function was further verified in hepatoma cells through ex vivo experiments. Finally, the antitumor activity and mechanism of Brilanestrant, a drug candidate, in HCC was preliminary investigated. In conclusion, this work demonstrated the antitumor activity of a drug candidate screened through our prognosis risk model in HCC, which provide a novel perspective in the area of drug exploration for cancer patients.

## Materials and methods

### Cell culture and reagents

HCC cells including HepG2, Huh7, PLC/PRF/5, and SK-Hep-1 were purchased from Cell Bank of Type Culture Collection of Chinese Academy of Sciences (Shanghai, China). Cells were cultured in DMEM supplemented with 10% FBS (SenBeiJia Biological Technology Co., Ltd, Nanjing, China) at 37 ℃ in a humidified chamber with 5% CO_2_. FastPure cell/tissue total RNA isolation kit (Cat# RC-112), HiScript II 1st strand cDNA synthesis kit (Cat# RC-312), and ChamQ SYBR qPCR Master Mix (Cat# Q-311) were purchased from Vazyme (Nanjing, China). Drugs including ML-323 (Cat# T1757), Adavosertib (Cat# T2077), Sepantronium bromide (Cat# T2111), Ceralasertib (Cat# T3338), Paclitaxel (Cat# T0968), and Brilanestrant (Cat# T5118) were purchased from Topscience (Shanghai, China). Ferrostatin-1 (Cat# HY-100579) was purchased from MedChemExpress (Shanghai, China). PE annexin V apoptosis detection kit (Cat# 559763) was purchased from BD Biosciences (Shanghai, China). Reactive oxygen species (ROS) assay kit (Cat# S0033S) was purchased from Beyotime (Shanghai, China). MTT assay kit (Cat# VIC292) was purchased from Vicmed (Xuzhou, China).

### Patient specimens

Tumor and matched para-carcinoma tissues were collected from HCC patients who had undergone surgical resection procedure at the Affiliated Hospital of Xuzhou Medical University, and restored at – 80 °C for further RNA extraction. All procedures in this study involving human participants were in accordance with the ethical standards of the institutional and/or national research committees and with the 1964 Declaration of Helsinki and its later amendments or comparable ethical standards. The use of human biological materials was approved by the Ethics Committee of Affiliated Hospital of Xuzhou Medical University (Permit Number: XYFY2023-KL246-01).

### Publicly available data acquisition

The raw data of RNA-sequencing (level 3) and corresponding clinical information of HCC patients were obtained from TCGA dataset (TCGA_LH, https://portal.gdc.cancer.gov) and ICGC dataset (ICGC_LH_JP, https://dcc.icgc.org/releases/current/Projects). The sequencing data of HCC patients were retrieved for further analysis and the samples without survival time were not included, which yield a number of samples from TCGA (LH_370, normal liver samples_50) and ICGC (LH_240, normal liver sampels_202). The gene expression data of normal liver samples were obtained from GTEx dataset (Version 8, https://www.gtexportal.org/home/datasets). The microarray data of GSE76427 was downloaded from GEO dataset (http://www.ncbi.nih.gov/geo), and normalized by the normalize quantiles function of preprocessCore package in R software, which yield 115 HCC and 52 normal liver samples.

### Selection of prognosis-related genes

The DEGs in HCC compared to normal liver samples were generated using limma package in R software with TCGA, ICGC, and GSE74627 datasets. The threshold was cut off with adjusted *p* values < 0.05 and | log2(fold change) |> 1. The survival analysis of DEGs between high and low expression patients was performed via Kaplan–Meier (KM) analysis using survival and survminer package in R software. The *p* value and hazard ratio (HR) with 95% confidence interval (CI) were calculated by log-rank tests and univariate Cox proportional hazards regression. The candidate prognosis-related genes were primarily selected relying on the intersection of DEGs, which occupied at the top 100 upregulated and downregulated expression in TCGA, ICGC, and GEO cohorts. In the meantime, the survival analysis including overall survival (OS), progression-free survival (PFS), disease-specific survival (DSS), and disease-free survival (DFS) of candidate prognosis-related genes should also be significant (*p* values < 0.05) for HCC patients. Finally, 11 genes were selected as prognosis-related genes for further analysis.

### Signature construction and expression verification of prognosis-related genes

The chromosomal positions of prognosis-related genes were visualized using OmicCircos package in R software. The mutation profiles in HCC patients were carried out with TCGA dataset, the mutation data were downloaded and visualized with maftools package in R software. The verification of prognosis-related genes at mRNA levels was performed with TCGA and GTEx datasets. Moreover, the validation of prognosis-related genes at protein levels was performed with CPTAC dataset at UALCAN data analysis portal (http://ualcan.path.uab.edu.). The representative immunohistochemistry images of prognosis-related genes at protein levels in normal and liver cancer tissues were obtained from HPA dataset (https://www.proteinatlas.org/).

### Quantitative polymerase chain reaction

The verification of prognosis-related genes at mRNA levels was also performed in our collected human specimens. In brief, total RNA was isolated from tumor and matched para-carcinoma tissues using a total RNA isolation kit according to manufacturer’s instructions. After synthesized cDNA with a synthesis kit, qPCR was performed using ChamQ SYBR qPCR Master Mix on Roche LightCycler® 480 system following manufacturer’s instructions. qPCR data was normalized to β-actin housekeeping control. Primer sequences were referenced from ORIGENE (https://www.origene.com). Relative gene expression values were calculated following 2^−ΔΔCt^ method^10^.

### Construction and validation of prognosis risk model

In order to establish prediction risk model with prognosis-related genes, TCGA_LH dataset was used to train the model, and ICGC_LH_JP dataset was chosen to externally validate the model. Primarily, 11 prognosis-related genes were subjected to multivariate Cox regression analysis to achieve the coefficients in R software. The risk-score formula was established as: risk score = ${\sum }_{i=1}^{11}(Expi\times Coei)$. The $Expi$ was the expression value of prognosis-related genes, and the $Coei$ was the coefficient of multivariate Cox regression analysis. The patients were divided into low- and high-risk groups based on the median risk score cutoff value. The KM analysis with log-rank test was used to compare overall survival between low- and high-risk groups. Receiver operating characteristic (ROC) analysis was performed to evaluate the accuracy of our risk model using survivalROC package in R software. Furthermore, the univariate and multivariate Cox proportional-hazards analyses were used to investigate the correlation between risk score and patients’ clinical characteristics. Then, a nomogram with c-index value was established based on risk score and patients’ clinical characteristics with survival and rms package in R software.

For external validation, the risk score of each patient was calculated with the expression level of each gene and the coefficients generated by training model. Then, the patients were stratified into low- and high-risk groups based on the median risk score cutoff value. The KM survival and ROC analyses were also performed as above described.

### Functional enrichment and pathway correlation analysis

The Kyoto Encyclopedia of Genes and Genomes (KEGG) and Gene Ontology (GO) including biological processes (BP), cellular components (CC), and molecular functions (MF) enrichment analyses on prognosis-related genes were performed with clusterProfiler package in R software on the platform Sangerbox^11,12^. A *p* value < 0.05 and false discovery rate (FDR) < 0.1 were considered statistically significant. Gene set enrichment analysis (GSEA) of significant biological pathways in low- and high-risk groups was carried out using multiGSEA package in R software. The validated gene signatures contributing to several pathways were primarily collected^13^, then gene set variation analysis (GSVA) package with 'ssgsea' method in R software was performed to calculate the correlation between gene expression and pathway. Furthermore, the correlation between risk score and pathway, as well as the correlation between gene and pathway score, was analyzed by Spearman correlation.

### Immune infiltration and checkpoint blockade analyses

The immune cell infiltration levels in individual HCC samples were quantified using single-sample gene set enrichment analysis (ssGSEA) with 'gsva' package in R software. The correlation between prognosis-related genes, risk score, and tumor infiltrating immune cells was evaluated with CIBERSORT algorithm^14^. The response of individual HCC samples to immune checkpoint blockade was predicted with tumor immune dysfunction and exclusion (TIDE) algorithm^15^.

### Drug sensitivity analysis

The half-maximal inhibitory concentration (IC_50_) area under curve (AUC) values of potential drug responses were predicted with pRRophetic package in R software based on gene expression proliferation of HCC patients^16^. The IC_50_ AUC values of each corresponding drug in low- and high-risk groups were compared under student’s *t* test. The correlation between risk score and predicted sensitivity values was calculated with Spearman correlation analysis. The drug candidates were selected according to a criteria of *p* values < 0.05 (students’ *t* test), log2(fold change) value <  − 0.10, and correlation coefficient with risk score <  − 0.50. Furthermore, cell toxicity of drug candidates was quantified in various human HCC cells inducing HepG2, Huh7, PLC/PRF/5, and Sk-Hep-1.

### Cell proliferation and colony formation analyses

Cells were seeded onto 96-well plates, then medium was replaced with drug solutions in the next day. After incubated for a further 48 h, cell viability was evaluated with an MTT assay kit following manufacturer’s instructions. Finally, IC_50_ values response curves were generated with GraphPad Prism 9.0 (GraphPad Software, Inc., USA). Colony formation analysis was performed following our previous study^17^. Briefly, cells were transferred onto 24-well plates, then treated with Brilanestrant (Bril, 30 μM) and/or Ferrostatin-1 (Ferr-1, 5 μM). After further cultured for one week, the cells were fixed with 4% formaldehyde and stained with 0.1% crystal violet.

### Cell apoptosis and ROS analysis

After treated with Bril (30 μM) for 48 h, cells were collected with trypsin and washed twice with pre-cold PBS. Then cells were stained with a PE annexin V apoptosis detection kit or ROS assay kit according to the manufacturer’s instructions. Finally, flow cytometry was used to detect cell apoptosis and ROS levels.

### Molecule docking

Three-dimensional (3D) crystallographic structure of human glutathione peroxidase 4 (GPX4) protein was retrieved from Protein Data Bank (PDB ID: 6HKQ)^18^. Molecular docking simulations were conducted to explore the binding interaction between GPX4 and Brilanestrant. The simulations utilized molecular operating environment (MOE) software (version 2020.09) according to a previously described method^19^.

### Etics apprval and consnt to partiipate

All procedures in this study involving human participants were in accordance with the ethical standards of the institutional and/or national research committees and with the 1964 Declaration of Helsinki and its later amendments or comparable ethical standards. All participants signed informed consent forms. The use of human biological materials was approved by the Ethics Committee of Affiliated Hospital of Xuzhou Medical University (Permit Number: XYFY2023-KL246-01).

## Results

### Identification of prognosis-related genes in HCC patients

**Figure 1:**
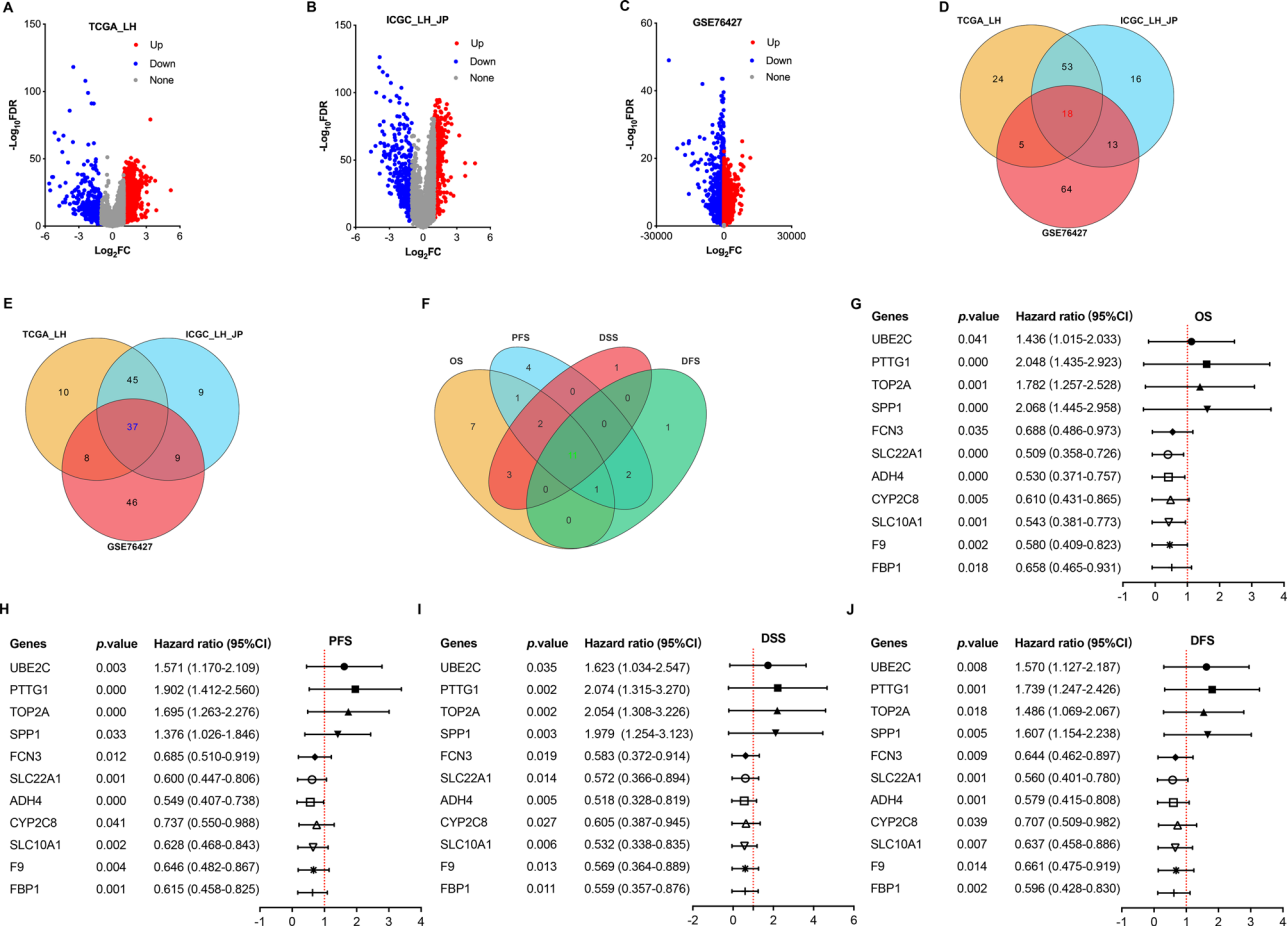
Identification of prognosis-related genes in HCC patients. (**A**–**C**) The volcano plot of DEGs in HCC samples compared to normal liver samples in TCGA_LH, ICGC_LH_JP, and GSE76427 datasets. Red dots represent upregulated genes, blue dots represent downregulated genes, and gray dots represent no changing ones. (**D**,**E**) The venn diagram of top 100 upregulated and downregulated expression genes in HCC. (**F**) The venn diagram of prognosis-related genes derived from the intersection top 100 upregulated and downregulated expression genes in HCC. (**G**–**J**) The *p*-value and hazard ratio of Kaplan–Meier survival analysis including OS, PFS, DSS and DFS for 11 prognosis-related genes in HCC patients based on TCGA dataset.

To compare gene expression levels between tumor and normal or adjacent tumor tissues in the liver, DEGs were explored within TCGA_LH, ICGC_LH_JP, and GSE76427 datasets using “limma” package in R software. The results showed that 2897 genes were identified as DEGs (2451 upregulated and 446 downregulated) in TCGA cohort (Fig. 1A), 966 genes were identified as DEGs (544 upregulated and 422 downregulated) in ICGC cohort (Fig. 1B), and 10,755 genes were identified as DEGs (8024 upregulated and 2731 downregulated) in GSE76427 cohort (Fig. 1C). Furthermore, the intersection of top 100 DEGs among TCGA, ICGC, and GSE76427 was performed to yield a total of 55 genes including 18 upregulated and 37 downregulated ones (Fig. 1D,E). After that, the survival analysis related to the 55 genes were performed with KM analysis based on TCGA dataset. The results showed that 11 genes (4 upregulated and 7 downregulated) were significantly associated with OS, PFS, DSS, and DFS in patients with HCC (Fig. 1F–J). Accordingly, UBE2C, PTTG1, TOP2A, SPP1, FCN3, SLC22A1, ADH4, CYP2C8, SLC10A1, F9, and FBP1 were selected as prognosis-related genes in HCC patients for further analysis.

### Variation and expression verification of 11 selected genes in HCC patients

**Figure 2:**
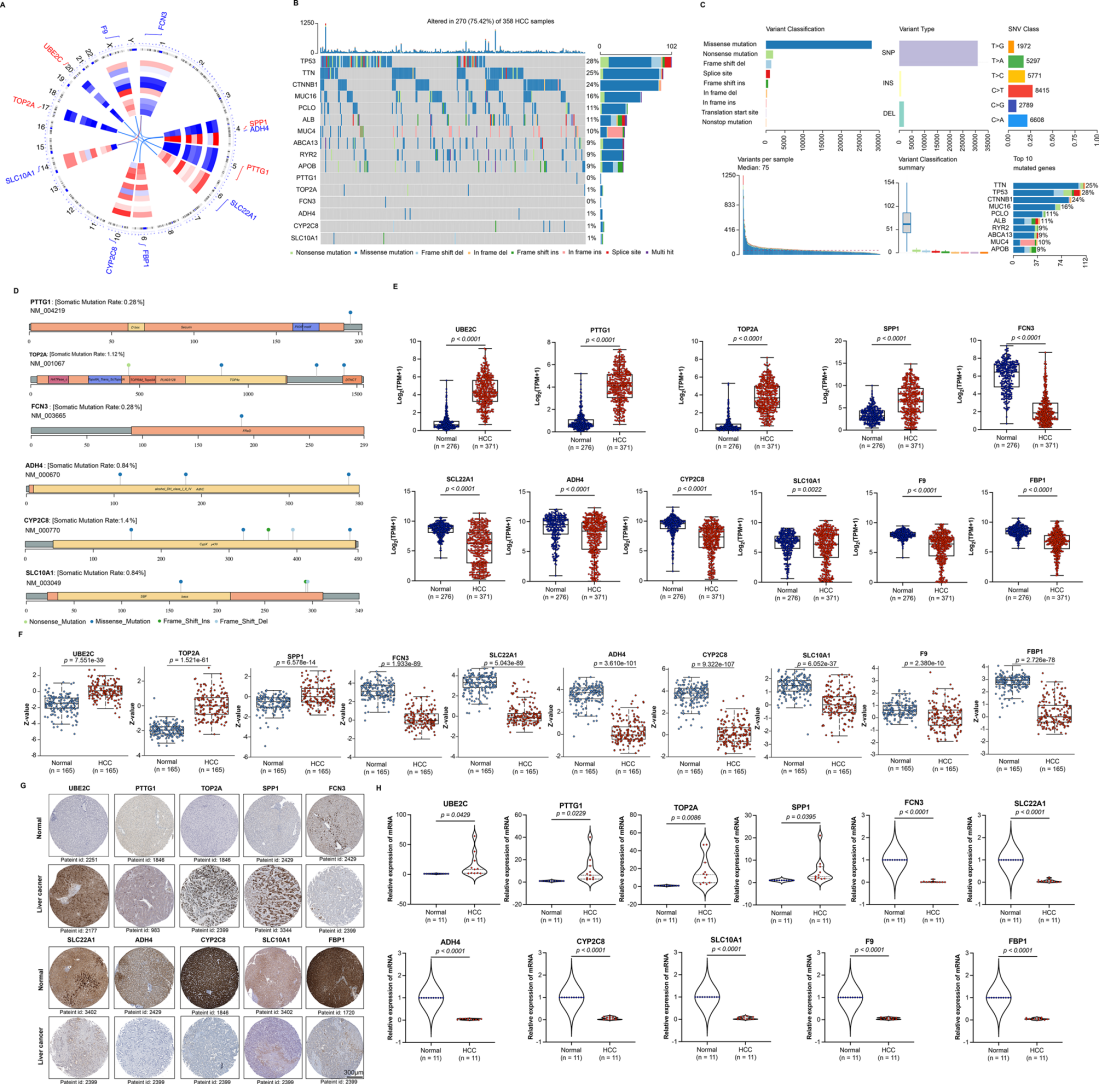
Signature construction and expression verification of 11 prognosis-related genes in HCC patients. (**A**) The chromosomal position visualization of 11 prognosis-related genes. (**B**) The mutation landscape in HCC samples based on TCGA dataset. The waterfall plot showed the mutation information of each gene (top 10 and 6 prognosis-related genes) in the sample. (**C**) The distribution of variants according to variant classification, type and single nucleotide variants (SNV) class in HCC samples. (**D**) The mutation distribution of 6 prognosis-related genes in HCC samples. (**E**) The expression verification of 11 prognosis-related genes in TCGA dataset (Normal: n = 50, HCC: n = 371) and GTEx dataset (Normal: n = 226) at mRNA levels. Data shown represent box and whisker plots (bar = median, box = interquartile range, whiskers = min to max all points), unpaired students’ *t* test was used to investigate the statistical difference. (**F**) The expression verification of 10 prognosis-related genes in CPTAC dataset (Normal: n = 165, HCC: n = 165) at protein levels. Data shown represent box and whisker plots (bar = median, box = interquartile range, whiskers = min to max all points), unpaired students’ *t* test was used to investigate the statistical difference. Data for PTTG1was not found. (**G**) The representative IHC staining images of 10 prognosis-related genes in liver cancer were from HPA dataset. Data for F9 was not found. (**H**) The expression verification of 11 prognosis-related genes using qPCR in our collected human specimens (Normal: n = 11, HCC: n = 11) at mRNA levels. Paired students’ *t* test was used to investigate the statistical difference.

To identify genome distribution of 11 prognosis-related genes, chromosome mapping was performed with genomic location of each gene. The results revealed a wide-genome distribution of 11 prognosis-related genes, which UBE2C, PTTG1, TOP2A, SPP1, FCN3, SLC22A1, ADH4, CYP2C8, SLC10A1 and FBP1 respectively located at chromosomes 20, 5, 17, 4, 1, 6, 4, 10, 14, and 9. F9 located at chromosome X (Fig. 2A). Furthermore, gene mutations in HCC patients were evaluated and visualized by using “maftools” package in R software based on TCGA dataset. The results showed that the top 10 high mutation frequency genes in HCC patients were TP53 (28%), TTN (25%), CTNNB1 (24%), MUC16 (16%), PCLO (11%), ALB (11%), MUC4 (10%), ABCA13 (9%), RYR2 (9%), and APOB (9%) (Fig. 2B). Moreover, missense mutation, single nucleotide polymorphism (SNP), and C > T single nucleotide variants (SNV) class were the predominant type of mutation or variant in HCC patients (Fig. 2C). Interestingly, among 11 prognosis-related genes, PTTG1 (0.28%), TOP2A (1.12%), FCN3 (0.28%), ADH4 (0.84%), CYP2C8 (1.4%), and SLC10A1 (0.84%) exhibited mutations, such as nonsense mutation, missense mutation, frame shift insertion, and frame shift deletion in HCC patients (Fig. 2B,D). However, there was no mutations for UBE2C, SPP1, SLC22A1, F9, and FBP1.

To confirm the expression of 11 prognosis-related genes at both mRNA and protein levels, TCGA, GTEx, CPTAC, and HPA datasets were retrieved and used for further analysis. Moreover, their expression levels were also investigated in our collected human specimens through qPCR. In TCGA and GTEx datasets, the results showed that UBE2C, PTTG1, TOP2A, and SPP1 were significantly upregulated in HCC samples compared to normal liver samples. Whereas, FCN3, SLC22A1, ADH4, CYP2C8, SLC10A1, F9, and FBP1 were significantly downregulated (Fig. 2E). Consistent with mRNA levels, the z-value corresponding to protein levels from CPTAC dataset of UBE2C, TOP2A, and SPP1 was higher. However, FCN3, SLC22A1, ADH4, CYP2C8, SLC10A1, F9, and FBP1 exhibited the opposite trend in HCC compared to normal liver tissues (Fig. 2F). Moreover, protein expression levels of 10 prognosis-related genes were also identified in human samples using IHC based on HPA dataset. The results showed that UBE2C, PTTG1, TOP2A and SPP1 were positive, FCN3, SLC22A1, ADH4, CYP2C8, SLC10A1, and FBP1 were negative in liver cancer tissues (Fig. 2G). Furthermore, our qPCR results revealed that UBE2C, PTTG1, TOP2A, and SPP1 were significantly upregulated. However, FCN3, SLC22A1, ADH4, CYP2C8, SLC10A1, F9, and FBP1 were significantly downregulated in HCC tissue compared to the matched para-carcinoma tissue (Fig. 2H). In a word, the expression levels of 11 prognosis-related genes were well verified in several datasets.

### Construction of a survival prediction risk model

**Figure 3:**
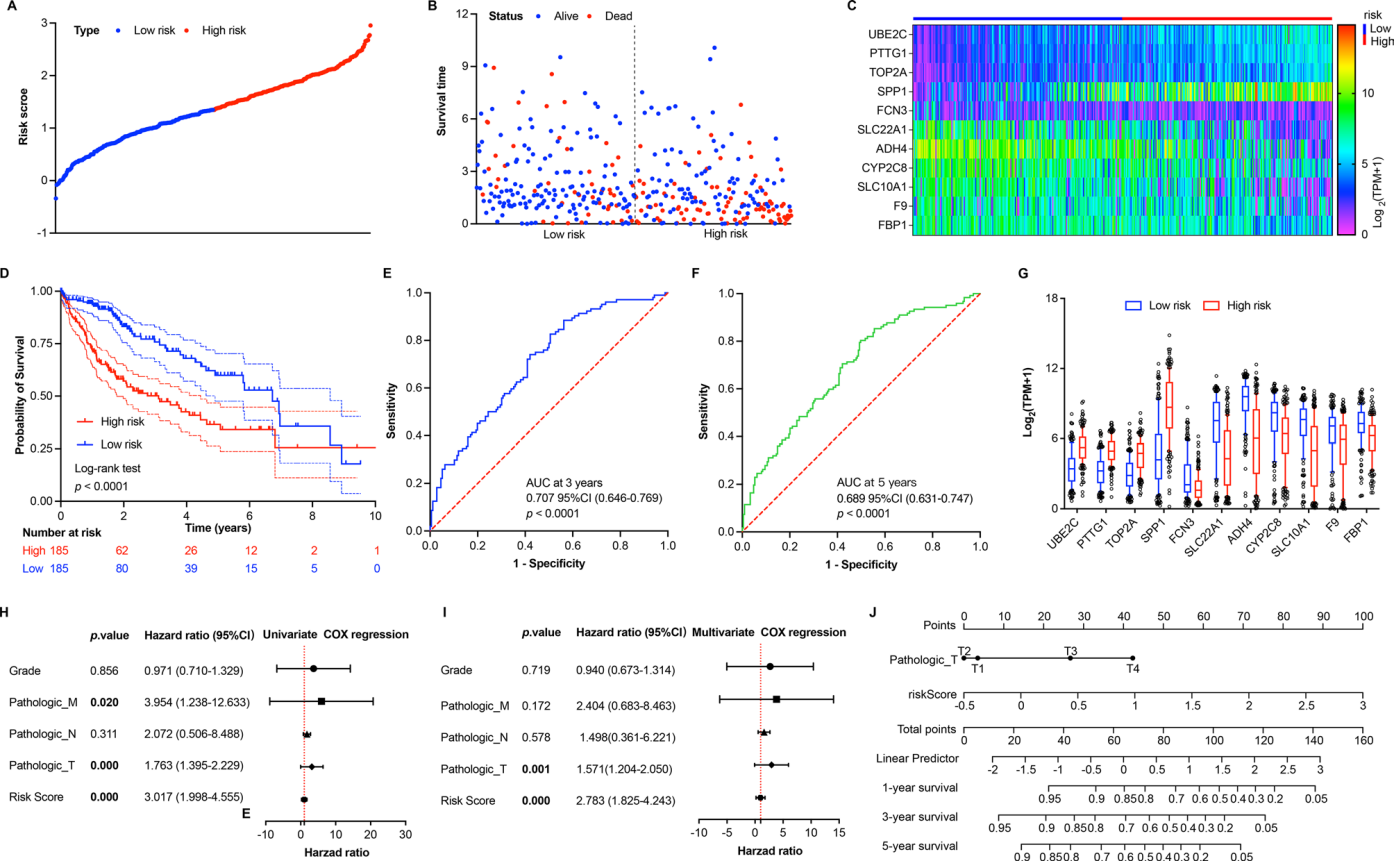
Construction of prediction risk model for HCC patients with TCGA dataset. (**A**) The distribution of risk score for each HCC patient based on prediction risk model. (**B**) The survival status of HCC patients based on risk score. (**C**) The heat map of 11 prognosis-related genes’ expression in low- and high-risk groups. (**D**) The KM overall survival analysis for HCC patients in low- and high-risk groups based on risk score. (**E**,**F**) The time-dependent ROC curves and AUC of 11 prognosis-related genes at 3- and 5-year based on prediction risk model. (**G**) The expression of 11 prognosis-related genes in low- and high-risk groups. Data shown represent box and whisker plots (bar = median, box = interquartile range, whiskers = 10–90 percentile), unpaired students’ *t* test was used to investigate the statistical difference between low- and high-risk groups, which generated a *p* value < 0.0001 for each gene. (**H**,**I**) The univariate and multivariate COX regression analyses of clinical characteristics related to HCC patients’ survival. (**J**) The Nomogram consisted of pathologic T stage and risk score for overall survival in HCC patients. The C-index of Nomogram is 0.748 with 95% CI (0.684–0.812) and* p* value = 2.56e−14.

The survival prediction risk model with 11 prognosis-related genes was established with multivariate Cox regression to generate a formula with risk score = $(-0.057)\times Exp(\text{UBE2C})+(0.2302)\times Exp(\text{PTTG1})+(0.0557)\times Exp(\text{TOP2A})+(0.0971)\times Exp(\text{SPP1})+(-0.0531)\times Exp(\text{FCN3})+(-0.0679)\times Exp(\text{SLC22A1})+(-0.0692)\times Exp(\text{ADH4})+(0.0462)\times Exp(\text{CYP2C8})+(-0.0138)\times Exp(\text{SLC10A1})+(0.1151)\times Exp(\text{F9})+(-0.0124)\times Exp(\text{FBP1})$ based on TCGA dataset. After calculated the risk score, HCC patients were divided into low-risk group (n = 185) and high-risk group (n = 185) based on the median risk score cutoff value (Fig. 3A). Survival status and time of each HCC patient in low- and high-risk groups were shown in Fig. 3B. Moreover, the relative expression levels of 11 prognosis-related genes for each HCC patient were shown in Fig. 3C. The KM survival analysis results showed that the overall survival time of HCC patients in low-risk group was significantly longer than that in high-risk group (Fig. 3D). Furthermore, the ROC was performed to evaluate the predictive efficiency of risk signature in 3- and 5-year survival rate. The results showed that the AUC was 0.707 with 95% CI (0.646–0.769) at 3-year and 0.689 with 95% CI (0.631–0.747) at 5-year, indicating a better predictive ability of our risk model (Fig. 3E,F). Furthermore, the expression levels of 11 prognosis-related genes in low- and high-risk groups were compared, showing that UBE2C, PTTG1, TOP2A, and SPP1 were upregulated; contrarily, FCN3, SLC22A1, ADH4, CYP2C8, SLC10A1, F9, and FBP1were downregulated in high-risk group (Fig. 3G).

Moreover, Cox regression analyses were performed to investigate the correlation between clinicopathological characteristics, risk score, and overall survival of HCC patients. Univariate Cox regression analysis results showed that pathologic M (HR 3.954, 95% CI 1.238–12.633, *p* = 0.020), pathologic T (HR 1.763, 95% CI 1.395–2.229, *p* = 0.000), and risk score (HR 3.017, 95% CI 1.998–4.555, *p* = 0.000) were significantly associated with overall survival of HCC patients based on TCGA dataset (Fig. 3H). Multivariate Cox regression analysis results showed that pathologic T (HR 1.571, 95% CI 1.204–2.050, *p* = 0.001), and risk score (HR 2.783, 95% CI 1.825–4.234, *p* = 0.000) were significantly associated with overall survival of HCC patients based on TCGA dataset (Fig. 3I). Then, a nomogram was constructed based on multivariate Cox repression analysis results to predict 1-, 3-, and 5-year survival rate for HCC patients (Fig. 3J). The c-index of our nomogram was 0.748 with 95% CI (0.684–0.812) corresponding to a *p* value of 2.56e-14, which suggested that our risk model had a good value for predicting the prognosis of HCC patients.

### Validation of the prediction risk model

**Figure 4:**
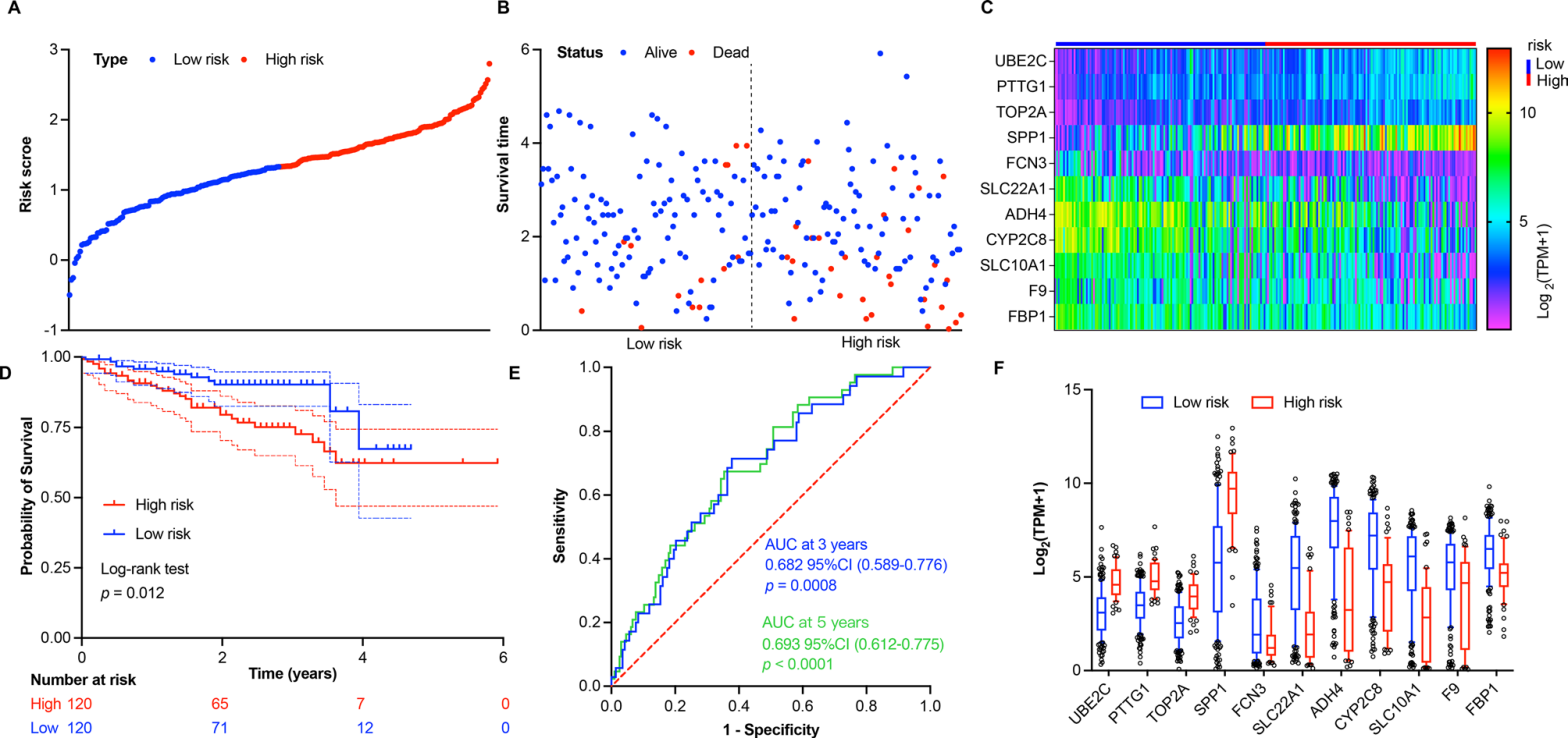
External validation of prediction risk model for HCC patients with ICGC dataset. (**A**) The distribution of risk score for each HCC patient based on prediction risk model. (**B**) The survival status of HCC patients based on risk score. (**C**) The heat map of 11 prognosis-related genes’ expression in low- and high-risk groups. (**D**) The KM overall survival analysis for HCC patients in low- and high-risk groups based on risk score. (**E**) The time-dependent ROC curves and AUC of 11 prognosis-related genes at 3- and 5-year based on prediction risk model. (**F**) The expression of 11 prognosis-related genes in low- and high-risk groups. Data shown represent box and whisker plots (bar = median, box = interquartile range, whiskers = 10–90 percentile), unpaired students’ *t* test was used to investigate the statistical difference between low- and high-risk groups, which generated a *p* value < 0.0001 for each gene.

To investigate the reliability of our prediction risk model, verification was performed with ICGC dataset. After calculated the risk score based on above mentioned formula, HCC patients were divided into low-risk group (n = 120) and high-risk group (n = 120) based on the median risk score (Fig. 4A). The survival status, survival time, and relative expression levels of 11 prognosis-related genes for each HCC patient in low- and high-risk groups were shown in Fig. 4B and 4C, respectively. Moreover, the KM survival analysis results showed that the overall survival rate of low-risk group was remarkedly higher than that in high-risk group (Fig. 4D). The AUC values were 0.682 with 95% CI (0.589–0.776) and 0.693 with 95% CI (0.612–0.775) at 3- and 5-year survival rate in HCC patients, respectively (Fig. 4E). In addition, the expression tendency of 11 prognosis-related genes in low- and high-risk groups was consistent with that in TCGA dataset (Fig. 4F). In summary, the external validation results based on ICGC dataset confirmed the reliability of our predictive risk model established with TCGA dataset.

### Enrichment analysis of 11 prognosis-related genes in HCC patients

**Figure 5:**
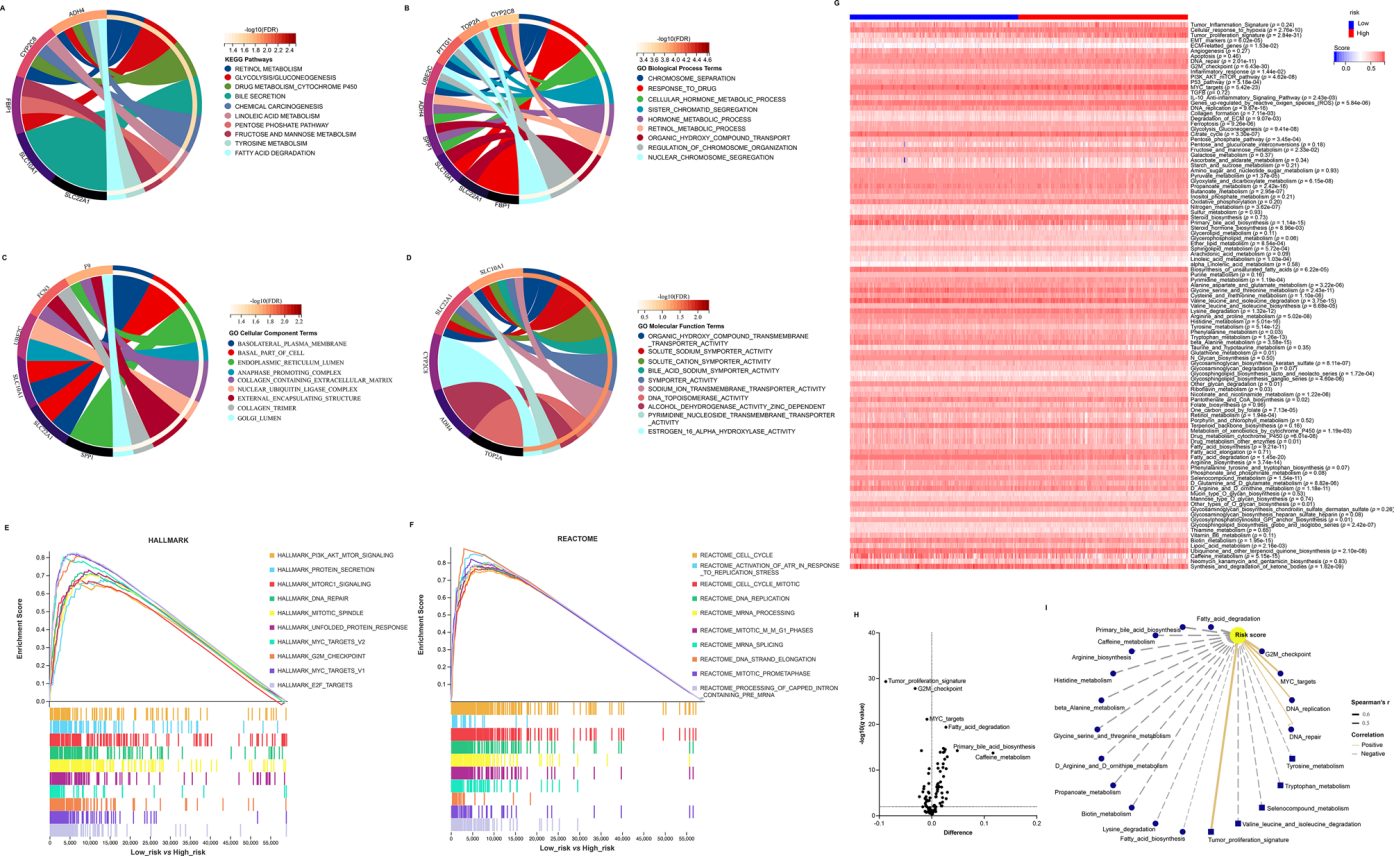
Functional enrichment and pathway correlation analyses associated with low- and high-risk in HCC patients. (**A**–**D**) The KEEG pathway, GO biological process, GO cellular component, and GO molecular function enrichment of 11 prognosis-related genes in HCC patients. (**E**,**F**) The GSEA enrichment analysis of hallmark and reactome associated with low- and high-risk in HCC patients based on TCGA dataset. (**G**) The heat map of pathways associated with low- and high-risk in HCC patients based on TCGA dataset. (**H**) The volcano plot of significant differential pathways associated with low- and high-risk (under unpaired students’ *t* test *p* value < 0.05) in HCC patients based on TCGA dataset. (**I**) The correlation network among significant differential pathways and risk score (|Spearman r |> 0.4 and *p* value < 0.05) in HCC patients based on TCGA dataset.

To explore the functions of 11prognosis-related genes, KEEG pathway and GO terms analyses were carried out using clusterProfiler package in R software. The results showed that retinol metabolism, glycolysis/gluconeogenesis, drug metabolism cytochrome P450, bile secretion, and chemical carcinogenesis were the key KEEG pathways (Fig. 5A). The biological process of cellular hormone metabolic process, chromosome separation, response to drug, and sister chromatid segregation were closely associated with the prognosis-related genes (Fig. 5B). The cellular component of basolateral plasma membrane, basal part of cell, endoplasmic reticulum lumen, and anaphase promoting complex were primarily enriched (Fig. 5C). The molecular functions were enriched in organic hydroxy compound transmembrane transporter activity, solute sodium symporter activity, solute cation symporter activity, bile acid sodium symporter activity, and so on (Fig. 5D).

Subsequently, GSEA was performed to identify the main enrichment pathways in two different risk groups. The results showed that the hallmark of PI3K_AKT_mTOR signaling, protein secretion, mTORC1 signaling, DNA repair, mitotic spindle, unfolded protein response, MYC targets v2, G2M checkpoint, MYC targets v1, and E2F targets were enriched in low-risk group (Fig. 5E). The reactome of cell cycle, activation of ATR in response to replication stress, cell cycle mitotic, DNA replication, mRNA processing, mitotic M M G1 phases, mRNA splicing, DNA strand elongation, mitotic prometaphase, and processing of capped intron containing pre-mRNA were also enriched in low-risk group (Fig. 5F).

Furthermore, the prognosis-related genes’ signatures contributing to collected pathways were carried out using GSVA analysis based on TCGA dataset^13^. The relative GSVA score of collected pathways for each HCC patient was shown in Fig. 5G. As expected, tumor proliferation signature, G2M checkpoint, and MYC targets were positively correlated with risk score. However, fatty acid degradation, primary bile acid biosynthesis, and caffein metabolism were negatively correlated with risk score (Fig. 5H,I).

### Immune response between low- and high-risk groups in HCC patients

**Figure 6:**
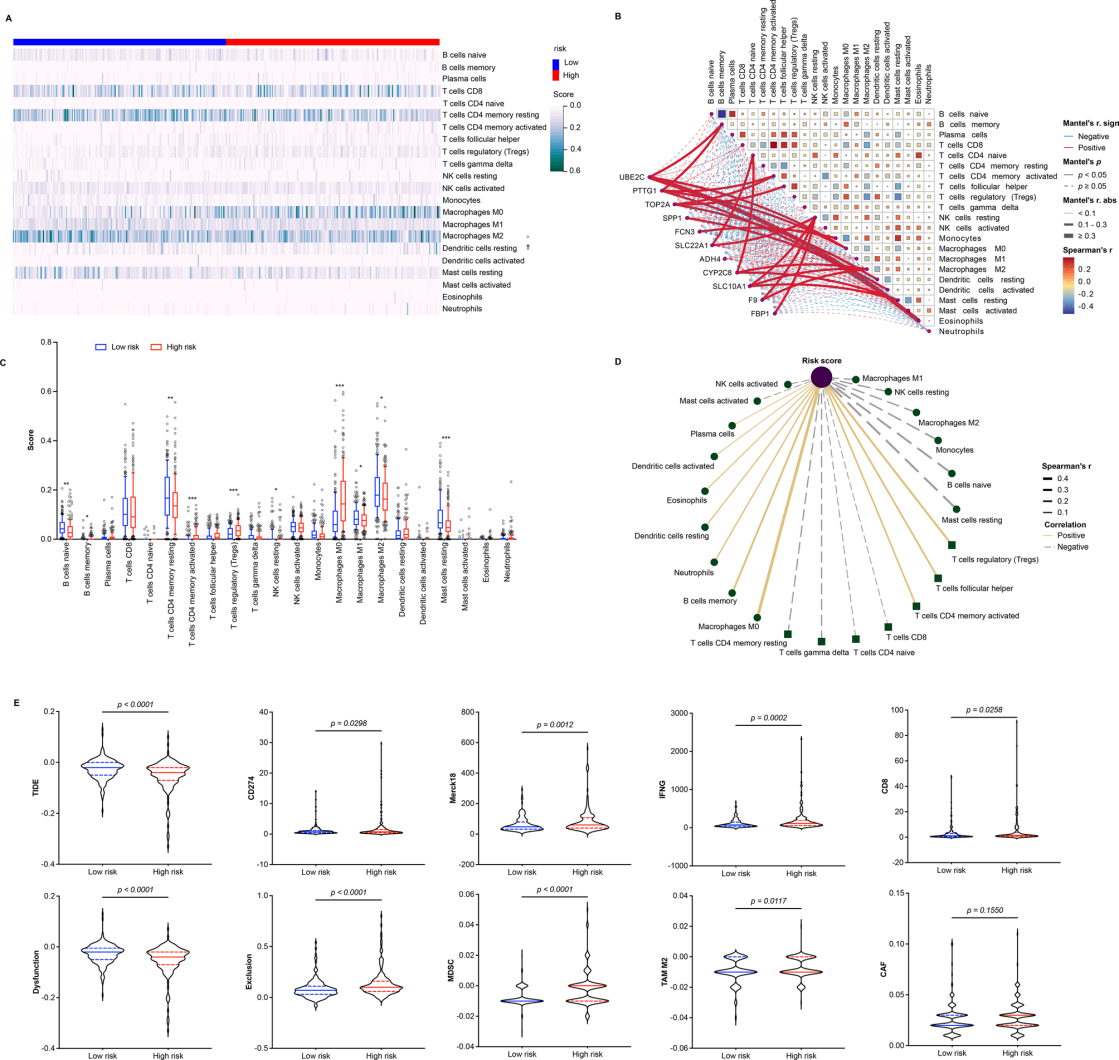
Immune cell infiltration and immunotherapy analyses associated with low- and high-risk in HCC patients. (**A**) The heat map of 22 kinds of immune infiltration cell associated low- and high-risk in HCC patients based on TCGA dataset. (**B**) The correlation of 22 kinds of immune infiltration cells (pairwise comparison with the Spearman’s correlation coefficients) and expression levels of 11 prognosis-related genes (curve width with the partial Mantel tests coefficients) in HCC patients based on TCGA dataset. (**C**) The comparison of immune infiltration cells between low- and high-risk groups. Data shown represent box and whisker plots (bar = median, box = interquartile range, whiskers = 10–90 percentile), unpaired students’ *t* test was used to investigate the statistical difference, the significant difference was denoted as *p < 0.05, **p < 0.01, ***p < 0.0001. (**D**) The correlation network among significant immune infiltration cells and risk score (|Spearman r|> 0.2 and *p* value < 0.05) in HCC patients based on TCGA dataset. (**E**) The response of low- and high-risk HCC patients to immune checkpoint blockade based on TIDE dataset. Unpaired students’ *t* test was used to investigate the statistical difference.

To investigate the response of low- and high-risk to immune infiltration cells, the CIBERSORT algorithm was used to evaluate the correlations based on TCGA dataset. The results showed that the risk score was positively correlated with T cells CD4 memory activated (Spearman r = 0.20, *p* = 0.0002), T cells follicular helper (Spearman r = 0.23, *p* = 6.56e−06), B cells memory (Spearman r = 0.25, *p* = 1.30e−06), T cells regulatory (Tregs)(Spearman r = 0.27, *p* = 1.56e−07), and macrophages M0 (Spearman r = 0.46, *p* = 1.73e−20). However, it was negatively correlated with monocytes (Spearman r = − 0.20, *p* = 0.0001), B cells naïve (Spearman r = − 0.26, *p* = 2.13e−07), and mast cells resting (Spearman r = − 0.29, *p* = 1.57e−08) (Fig. 6A,D). Moreover, the expression of UBE2C was positively correlated with eosinophils (Mantel r = 0.38, *p* < 0.05), B cells memory (Mantel r = 0.18, *p* < 0.05), and macrophages M0 (Mantel r = 0.17, *p* < 0.05). The expression of TOP2A was positively correlated with dendritic cells resting (Mantel r = 0.29, *p* < 0.05) and T cells follicular helper (Mantel r = 0.16, *p* < 0.05). The expression of PTTG1 was positively correlated with B cells memory (Mantel r = 0.20, *p* < 0.05), macrophages M0 (Mantel r = 0.18, *p* < 0.05), and eosinophils (Mantel r = 0.15, *p* < 0.05). The expression of SLC10A1 was positively correlated with mast cells resting(Mantel r = 0.18, *p* < 0.05) (Fig. 6B). In addition, B cells naïve, T cells CD4 memory resting, NK cells resting, macrophages M1, macrophages M2, and mast cells resting were significantly more activated in low-risk group compared to high-risk group. However, B cells memory, T cells CD4 memory activated, T cells regulatory (Tregs), and macrophages M0 were significantly more activated in high-risk group compared to low-risk group (Fig. 6C).

Furthermore, to predict the response of low- and high-risk to immune checkpoint blockade (ICB), TIDE score of HCC patients was calculated based on TCGA dataset. Subsequently, the comparison between low- and high-risk group was performed. The results showed that the TIDE and T cell dysfunction scores were significantly higher in low-risk group than that in high-risk group (Fig. 6E). Besides, other markers including CD274, Merck18, IFNG, CD8, T cell exclusion, MDSC, and TAM M2 were significantly more activated in high-risk group than that in low-risk group (Fig. 6E).

### Drug candidate prediction and verification for HCC

**Figure 7:**
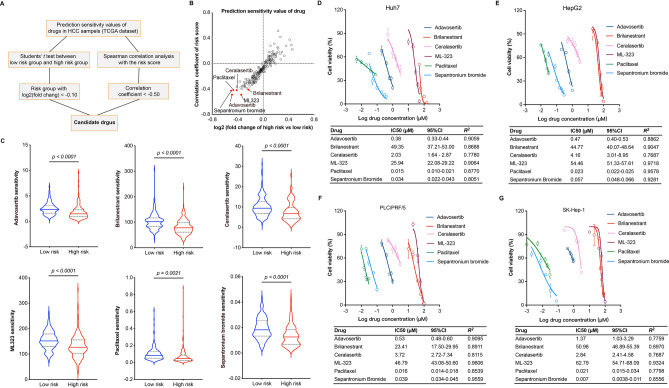
Drug sensitivity signature prediction and verification in HCC. (**A**) The workflow of drug sensitivity prediction in HCC patients based on TCGA dataset. (**B**) The scatterplot of predicted sensitivity of drug. The x axes represent log2 fold change of mean predicted sensitivity value of each drug in high-risk group *versus* that in low-risk group, the y axes represent the Spearman correlation coefficients between predicted sensitivity value of each drug and risk score in HCC patients. The drug candidates were marked in red color. (**C**) The sensitivity (IC_50_ AUC value) comparison of drug candidates in low- and high-risk groups, unpaired students’ *t* test was used to investigate the statistical difference. (**D**–**G**) The cell survival curve and IC_50_ value for HCC cells including Huh7, HepG2, PLC/PRF/5, and SK-Hep-1 after treated for 48 h with drug candidates. Data are presented as mean ± SEM in cell survival curve, IC_50_ values are presented with 95% CI and R squared value.

To search effective drug candidates for HCC, a screening criterion was carried out following previous studies’ descriptions based on our predictive risk model^16,20^. Primarily, drug sensitivity was predicted using pRRophetic package in R software based on TCGA dataset. Then, log2 fold change of mean predicted sensitivity value of drug in high-risk group versus that in low-risk group less than − 0.10 and the correlation coefficients of risk score less than − 0.50 were set as cut-off value to predict drug candidates (Fig. 7A). Eventually, six drug candidates including Adavosertib, Brilanestrant, Ceralasertib, ML-323, Paclitaxel, and Sepantronium bromide were predicted (Fig. 7B). Interestingly, the IC_50_ AUC values of them were remarkedly lower in high-risk group than that in low-risk group (Fig. 7C).

In order to evaluate the inhibitory effects of drug candidates on HCC, hepatoma cells including Huh7, HepG2, PLC/PRF/5, SK-Hep-1 were selected for further investigations. After treated 48 h with individual drug candidates under indicated concentrations, the IC_50_ values were calculated based on cell viability. The results showed that Paclitaxel and Sepantronium bromide exhibited strongest inhibitory effects to hepatoma cells corresponding to the lowest IC_50_ value. Moreover, Adavosertib and Ceralasertib showed half inhibitory effects to hepatoma cells around 0.5 ~ 4.0 μM. However, Brilanestrant and ML-323 occupied a high concentration for half inhibitory effects to hepatoma cells (Fig. 7D–G). Interestingly, reports have previously revealed the inhibitory effects of drug candidates on HCC without Brilanestrant^21–25^, which was selected for further analysis.

### Ferroptosis is the potential target of Brilanestrant in HCC

**Figure 8:**
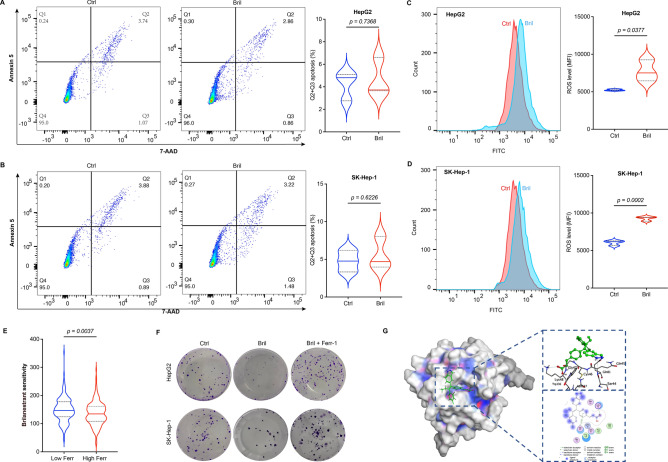
Ferroptosis is the potential target of Brilanestrant in HCC. (**A**,**B**) The cell apoptosis analysis in HepG2 and SK-Hep-1 cells after treated for 48 h with 30 μM of Brilanestrant (Bril). (**C**,**D**) The ROS detection in HepG2 and SK-Hep-1 cells after treated for 48 h with 30 μM of Bril. (**E**) The sensitivity (IC_50_ AUC value) comparison of Bril in low- and high-ferroptosis associated patients with HCC. (**F**) The representative images of colony formation in HepG2 and SK-Hep-1 cells after treated with Bril (30 μM) and Ferrostatin-1 (Ferr-1, 5 μM). (**G**) Molecular docking between Bril and GPX4. The graph includes the 3D conformation and 2D depiction of interactions. The binding free energy predictions of Bril to GPX4 was − 4.7 kcal/mol. All in vitro experiments were performed in triplicate, and unpaired students’ *t* test was used to investigate the statistical difference.

In order to clarify the molecular mechanism of Brilanestrant in inhibiting HCC cells, cell apoptosis that is a normal process of cell death in cancer induced by drugs was investigated following Brilanestrant (Bril) treatment. Our results showed that Bril treatment (30 μM for 48 h) could not induce apoptosis in hepatoma cells (Fig. 8A,B). Interestingly, Bril treatment could significantly increase intracellular ROS levels (Fig. 8C,D). Furthermore, the IC_50_ AUC value of Bril was remarkedly lower in high-ferroptosis associated HCC patients than that in low ones (Fig. 8E). Surprisingly, Ferrostatin-1 (Ferr-1, a potent and selective ferroptosis inhibitor) treatment could effectively reverse Bril induced cell death in HCC cells (Fig. 8F). Moreover, molecular docking simulations have revealed a binding affinity between Bril and GPX4, which is quantified by a free energy of − 4.7 kcal/mol. The analysis has identified a hydrogen bond wherein the carboxyl oxygen of Bril forms an interaction with the lysine residue Lys48 of GPX4 (Fig. 8G). This specific interaction is postulated to contribute for the modulation of the biological activity of GPX4.

## Discussion

Over the past few decades, bioinformatics has been widely applied in the area of HCC research. Currently, a series of studies have developed the prognosis prediction model for HCC patients based on bioinformatics analysis. Recently, He Q. et al. constructed a prognostic and diagnostic model related to transcatheter arterial chemoembolization (TACE) refractoriness for HCC patients^9^. Yang T. et al. established a model for predicting the prognosis and immune status of HCC patients^26^. Here, we developed a novel prediction model with 11 prognosis-related genes in liver cancer, which could significantly predict the clinical outcome of cancer patients. Importantly, our prediction model could also effectively evaluate the immunotherapy and drug treatment responsiveness for the patients.

In our research, to explore factors contributing to the malignancy of liver cancer, DEGs were identified in TCGA_LH, ICGC_LH, and GSE76427 datasets. The intersection of top 100 DEGs in three datasets were performed to yield 55 genes including 18 upregulated and 37 downregulated. Using TCGA dataset, 11 genes (UBE2C, PTTG1, TOP2A, SPP1, FCN3, SLC22A1, ADH4, CYP2C8, SLC10A1, F9, and FBP1) were significantly identified to associate with OS, PFS, DSS and DFS in HCC patients. In the present study, gene signature analysis showed that the 11 genes without F9 widely located at different euchromosomes, 6/11 genes exhibited low frequency mutations including PTTG1, TOP2A, FCN3, ADH4, CYP2C8, and SLC10A1(Fig. 2).

In previous studies, ubiquitin-conjugating enzyme E2C (UBE2C), pituitary tumor transforming gene 1 (PTTG1), topoisomerase II alpha (TOP2A), and secreted phosphoprotein 1 (SPP1) have been recognized as oncogenes promoting the progression of HCC. Here, the expression level of them were also well confirmed in public datasets and our collected human specimens (Fig. 2). It has been revealed that UBE2C could enhance cell proliferation, migration, invasion, and drug resistance in HCC cells^27,28^. PTTG1 is remarkedly overexpressed in HCC samples and involved in angiogenesis, proliferation, metastasis and metabolism reprogramming of HCC, which serves as a therapeutic and diagnostic target^29–31^. Overexpression TOP2A is positively correlated with poor prognosis of HCC. It could enhance the proliferation, metastasis, invasion and epithelial-mesenchymal transition process of HCC^32–34^. In addition, expression of SPP1 is closely associated with tumor cell evolution and microenvironmental reprogramming. It functions as an enhancer of cell growth in HCC^35,36^.

Whereas, to be tumor suppressors, ficolin 3 (FCN3), cytochrome P450 family 2 subfamily C member 8 (CYP2C8), and fructose-1,6-biphosphatase (FBP1) have been reported to be downregulated in HCC and inhibited the progression of HCC. Ma D. et al. revealed that high-expression of FCN3 was positively associated with a good prognosis for liver cancer patient. It could induce cell apoptosis and inhibit cell proliferation in HCC^37^. CYP2C8 has been revealed to enhance the anticancer activity of sorafenib and inhibit HCC cell malignant phenotypes including proliferation, clonality, migration, invasion and cell cycle^38^. Moreover, FBP1 appears to be a tumor suppressor in HCC progression though negatively regulating the Warburg effect^39^. In addition, studies have demonstrated that the expression of solute carrier family 22 member 1 (SLC22A1), alcohol dehydrogenase 4 (ADH4), solute carrier family 10 member 1 (SLC10A1), and coagulation factor IX (F9) was related to the progression and survival of HCC^6,40–42^. However, there is no reference regarding the integration of these genes for the prognosis of patients with HCC.

Herein, we created a 11 prognosis-related genes prediction risk model using machine learning algorithm based on TCGA dataset. Satisfactorily, the model could well divide HCC patients into low- and high-risk groups. Moreover, the patients with low-risk score showed a significant better overall survival probability than high-risk ones. The AUC values of 3- and 5-year were respectively 0.707 and 0.689 (*p* < 0.0001) (Fig. 3). Recently, numerous models were established to evaluate the prognosis of HCC patients. Gao S. et al. developed a prognostic model utilizing 10 genes, with corresponding AUC values of 0.67 and 0.66 for 3- and 5-year time points, respectively^43^. Moreover, Gao Q. et al. built a prognostic model with 8 genes, achieving AUC values of 0.645 and 0.630 at 3- and 5-year, respectively^44^. According to multivariate Cox repression analyses results, a nomogram model with c-index value of 0.748 was further established to predict 1-, 3-, and 5-year survival of HCC patients (Fig. 3), which is higher than previous ones^26,45^. Moreover, the scoring system was also externally validated with ICGC dataset (Fig. 4). All of which indicate that our prognosis risk model has a better predictive power, suggesting its application value in clinic and practice.

Studies show that sustaining proliferative signaling and deregulating cellular metabolism are the hallmarks of cancer, and lipid metabolism is aberrantly activated in most cancers^46,47^. Here, our results revealed that the risk score was positively correlated with tumor proliferation signature, but negatively correlated with fatty acid degradation (Fig. 5), indicating that HCC patients with high-risk score maintain stronger malignant progression. In other words, these findings further prove the reliability of our model in another way.

Liver reserves abundant immune cells that participate in tumorigenesis, metastasis, and drug resistance of cancer^48^. Macrophages M0 is the only resting state of macrophages. It has been reported that high infiltration of macrophages M0 showed a poor overall survival in HCC patients^49,50^. In this work, our results showed that the risk score was most positively correlated with infiltrated macrophages M0 (Fig. 6), revealing that high infiltration of macrophages M0 is unfavorable factor for HCC patients. Mast cells are the major contributors to allergic disease, also associated with reduced survival rates in colorectal cancer^51^. Here, we found that mast cells resting was most negatively associated with the risk score (Fig. 6), indicating that the unfavorable role of mast cells in HCC. The primary mechanism of tumor immune evasion is to induce T cell dysfunction and prevent T cell infiltration in tumors, TIDE score is a suitable rule to investigate factors underlying this mechanism^15^. In our study, TIDE score was significantly higher in low-risk group, indicating that HCC patients with low-risk score could receive a poor immune checkpoint inhibition treatment.

At present, sorafenib and lenvatinib are approved by U. S. Food and Drug Administration as the first-line option for targeted therapy of advanced liver cancer^52^. However, novel additional therapeutics are required to explore for HCC patients due to drug-resistance. In this study, a prediction method was developed to screen potential drug candidates using integrating patients’ risk score and drug IC_50_ values. Accordingly, six potential drugs were identified as candidates for HCC treatment, drug inhibitory activity to HCC cells was investigated through ex vivo experiments (Fig. 7). Interestingly, previous results exhibited the inhibitory effects of adavosertib, ceralasertib, ML-323, paclitaxel, and sepantronium bromide to HCC cells^21–25^. It seems reasonable to conclude that our drug screening model is reliable and effective. Brilanestrant, an orally bioavailable selective estrogen receptor degrader, has strong anti-tumor activity in human breast cancer both in vitro and in vivo^53,54^. Moreover, it has been evaluated in Phase II clinical studies in breast cancer^55^. However, rare studies reveal its anti-tumor activity in liver cancer. Here, our results showed that Brilanestrant could obviously increase intracellular ROS level, which is a pivotal biomarker of ferroptosis^56^. Importantly, Ferrostatin-1 (the ferroptosis inhibitor) could effectively reverse Brilanestrant induced cell death in HCC cells. In addition, Brilanestrant is able to bind to and inhibit the activity of GPX4 (Fig. 8), which is the center molecular of ferroptosis regulation^56,57^. In summary, these results indicate that ferroptosis is a potential target of Birlanestrant in HCC, but the mechanism should be further elucidated in the future.

However, some limitations should be recognized in this study. First, the prognostic predictive capability of our risk model should be further validated by more external datasets. Second, our model’s function for identifying the cancer stage of HCC should be developed in the future. Moreover, further in vitro and in vivo experiments should be performed to investigate the roles of prognosis-related genes and drug candidates in the progression of HCC. Taken together, more studies are needed to verify our model.

## Conclusion

In conclusion, our research developed and validated a prediction risk model of HCC based on 11 prognosis-related genes. This risk model could effectively evaluate the overall survival, immune cell infiltration, and immunotherapy response of HCC patients. Importantly, a potential anti-liver cancer drug candidate, Brilanestrant, was identified relying on our risk model and primarilyvalidated through in vitro experiments. Overall, our findings have great significance for prognosis and treatment of the patients with HCC.

## Data Availability

The datasets analyzed in this study are available in the TCGA, ICGC, GEO, GTEx, HPA, and CPTAC repository.

## Abbreviations

3D: Three-dimensional
ADH4: Alcohol dehydrogenase 4
BP: Biological processes
Bril: Brilanestrant
CAF: Cancer associated fibroblasts
CC: Cellular components
CD: Cluster of differentiation
CPTAC: Clinical proteomic tumor analysis consortium
CYP2C8: Cytochrome P450 family 2 subfamily C member 8
DEGs: Differentially expressed genes
DFS: Disease-free survival
DSS: Disease-specific survival
F9: Coagulation factor IX
FBP1: Fructose-1,6-biphosphatase
FCN3: Ficolin 3
FDR: False discovery rate
GEO: Gene expression omnibus
GO: Gene Ontology
GPX4: Glutathione peroxidase 4
GSEA: Gene set enrichment analysis
GSVA: Gene set variation analysis
GTEx: Genotype tissue expression
HCC: Hepatocellular carcinoma
HPA: Human protein atlas
ICGC: International cancer genome consortium
IFNG: Interferon gamma
KEEG: Kyoto encyclopedia of genes and genomes
KM: Kaplan–Meier
MDSC: Myeloid-derived suppressor cells
MF: Molecular functions
MOE: Molecular operating environment
OS: Overall survival
PFS: Progression-free survival
PTTG1: Pituitary tumor transforming gene 1
ROC: Receiver operating characteristic
SLC10A1: Solute carrier family 10 member 1
SLC22A1: Solute carrier family 22 member 1
SPP1: Secreted phosphoprotein 1
ssGSEA: Single-sample gene set enrichment analysis
TAM: Tumor associated macrophage
TCGA: The cancer genome atlas
TIDE: Tumor immune dysfunction and exclusion
TOP2A: Topoisomerase II alpha
UBE2C: Ubiquitin-conjugating enzyme E2C
